# The Potential Role of the NLRP3 Inflammasome Activation as a Link Between Mitochondria ROS Generation and Neuroinflammation in Postoperative Cognitive Dysfunction

**DOI:** 10.3389/fncel.2019.00073

**Published:** 2019-02-21

**Authors:** Penghui Wei, Fan Yang, Qiang Zheng, Wenxi Tang, Jianjun Li

**Affiliations:** ^1^Department of Anesthesiology, Qilu Hospital of Shandong University, Qingdao, China; ^2^Department of Anesthesiology, Cheeloo College of Medicine, Shandong University, Jinan, China

**Keywords:** postoperative cognitive dysfunction, NLRP3 inflammasome, mitochondria-derived reactive oxygen species, Interleukin-1β, neuroinflammation, microglia, hippocampus

## Abstract

Postoperative cognitive dysfunction (POCD) is commonly observed in perioperative care following major surgery and general anesthesia in elderly individuals. No preventive or interventional agents have been established so far. Although the role of interleukin-1β (IL-1β)-mediated neuroinflammation following surgery and anesthesia is strongly implicated in POCD, the exact mechanism of action remains to be explored. Growing evidence has shown that mitochondria-derived reactive oxygen species (mtROS) are closely linked to IL-1β expression through a redox sensor known as the nod-like receptor pyrin domain-containing 3 (NLRP3) inflammasome. Therefore, we hypothesize that the mechanisms underlying POCD involve the mtROS/NLRP3 inflammasome/IL-1β signaling pathway. Furthermore, we speculate that cholinergic anti-inflammatory pathway induced by α7 nicotinic acetylcholine receptor (a7nAChR) may be the potential upstream of mtROS/NLRP3 inflammasome/IL-1β signaling pathway in POCD. For validating the hypotheses, we provide experimental plan involving different paradigms namely; microglial cells and behavioral studies. The link between mtROS, the NLRP3 inflammasome, and IL-1β within and between these different stages in combination with mtROS and NLRP3 inflammasome agonists and inhibitors could be explored using techniques, such as knockout mice, small interference ribonucleic acid, flow cytometry, co-immunoprecipitation, and the Morris Water Maze test. We conclude that the NLRP3 inflammasome is a new preventive and therapeutic target for POCD.

## Introduction

Postoperative cognitive dysfunction (POCD) is a highly prevalent condition with significant effects on the prognosis of elderly patients undergoing surgery, experiencing problems with memory, concentration, information processing, language comprehension, and social integration that can last for months or may even be permanent (Leslie, 2017). POCD reportedly occurs in 25% to 40% of elderly patients undergoing cardiac surgery, non-cardiac surgery, and even minor non-invasive procedures under sedation, such as coronary angiography, across different studies (Evered et al., 2011). The potential risk factors of POCD include advanced age, lower education levels, carrying the APOE4 genotype, alcohol abuse and premedication (Skvarc et al., 2018; Xie et al., 2018). Of note, preoperative treatment with anticholinergic medications (e.g., atropine and scopolamine) or medications with anticholinergic properties (e.g., tricyclic antidepressants and benzodiazepines) have been demonstrated to be associated with increasing risk of POCD in recent years (Wang et al., 2014). Shoair et al. (2015) conducted a subgroup analysis of 69 patients aged 65 years or older, and they found that the incidence of POCD in the patients receiving anticholinergic or sedative-hypnotic drug at home prior to surgery was three times greater than patients without these drug (users 28% vs. nonusers 9.1%). A large longitudinal follow up of patients who had POCD 1 week or 3 months following surgery showed significantly higher mortality [hazard ratio, 1.63 (95% confidence interval, 1.11–2.38); *P* = 0.01, adjusted for sex, age, and cancer] and risk of leaving the labor market prematurely [hazard ratio, 2.26 (1.24–4.12); *P* = 0.01], and greater dependency on social security [prevalence ratio, 1.45 (1.03–2.04); *P* = 0.03] (Steinmetz et al., 2009). As the aging population requiring more surgeries is increasing, POCD is expected to become epidemic.

Inflammation and immune activation are the key mechanism of POCD. Surgery and anesthesia unleash a body-wide inflammation in the elderly, and then peripheral inflammatory cytokines can compromise the integrity of the blood brain barrier (BBB), allowing for increased infiltration of inflammatory factors and macrophages into brain (Terrando et al., 2011; Leslie, 2017). Although current theories regarding the mechanisms underlying POCD highlight the role of neuroinflammation in the hippocampus (Skvarc et al., 2018), the exact cascade remains elusive. interleukin (IL)-1β-mediated neuroinflammation in the hippocampus plays a pivotal role in surgery-induced cognitive dysfunction; both in mice pretreated with IL-1 receptor antagonist and knocked out IL-1 receptor (IL-1R^−/−^), the cognitive impairment induced by surgical trauma was effectively attenuated (Cibelli et al., 2010). Our previous study has also confirmed the critical role of hippocampal IL-1β in the development of POCD (Li et al., 2014; Wei et al., 2018). However, the mechanism by which surgical stress and anesthesia induce production of IL-1β in association with POCD remains unknown.

The nod-like receptor pyrin domain-containing 3 (NLRP3) inflammasome, composed of NLRP3 protein, adapter protein apoptosis-associated speck-like protein (ASC), and pro-caspase-1, is a pivotal upstream target that controls IL-1β cleavage and secretion by the active caspase-1 (Schroder and Tschopp, 2010). Recent studies have revealed that isoflurane-induced cognitive impairment was associated with high levels of NLRP3 in the hippocampus of aged mice and the impairment was reversed by the inhibition of NLRP3-caspase-1 pathway (Wang et al., 2018). Studies have also reported that inhibitors of the NLRP3 inflammasome [e.g., MCC950 and ketone metabolite β-hydroxybutyrate (BHB)] inhibited NLRP3-induced ASC oligomerization and IL-1β expression in systemic macrophages and brain mononuclear cells (Coll et al., 2015; Youm et al., 2015). A recent study demonstrated that MCC950 improved cognitive function in Alzheimer’s disease (AD) by clearance of amyloid β (Aβ)_1–40_ and Aβ_1–42_ in apolipoprotein (APP)/presenilin 1 (PS1) mice. The study further showed that the impact of MCC950 on Aβ pathology resulted from its ability to block NLRP3 inflammasome activation in microglia (Dempsey et al., 2017). Therefore, the NLRP3 inflammasome may be a viable target to interrupt the pathogenesis of POCD. Furthermore, exposure to anesthetics may impair mitochondria and potentiate oxidative damage to neurons (Skvarc et al., 2018). The mitochondria are potent activators of the immune system through their ability to generate reactive oxygen species (ROS), which damage the mitochondrial DNA (mtDNA) and interact with the NLRP3 inflammasome during the inflammatory response (Kim et al., 2015; Liu K. et al., 2017). Evidence supported the critical role of mitochondrial ROS (mtROS) in NLRP3 inflammasome activation (Zhou et al., 2011). Zhou et al. (2011) showed that ROS generated by mitochondria having reduced membrane potential could lead to NLRP3 inflammasome activation and addition of the complex I inhibitor (rotenone) resulted in the loss of ROS generation and inflammasome activation. Moreover, a specific mitochondria ROS scavenger, the mito-TEMPO (500 μM), abrogated mtROS release, inhibited NLRP3 inflammasome activation and reduced the up-regulation of IL-1β and IL-18 induced by ethanol or lipopolysaccharide (LPS)/ATP (Alfonso-Loeches et al., 2014). Therefore, we speculate that mtROS/NLRP3 inflammasome-induced IL-1β activation in the hippocampus may play a critical role in the development of POCD, and this signaling pathway may consequently become an attractive drug target.

## The Hypothesis

**Figure 1:**
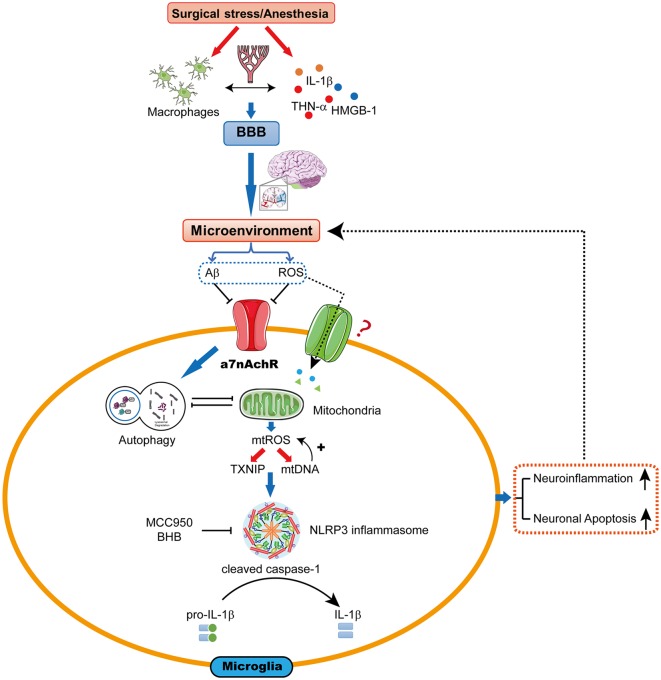
The proposed biological mechanisms for microglia mitochondrial reactive oxygen species (mtROS)/nod-like receptor pyrin domain-containing 3 (NLRP3) inflammasome-induced interleukin-1β (IL-1β) activation in the hippocampus leading to cognitive dysfunction following surgery and anesthesia. Surgical stress and anesthesia, through mechanisms that include inhibition of α7 nicotinic acetylcholine receptor (α7nAChR)-induced cholinergic anti-inflammatory pathway and autophagy, result in mitochondrial damage. The damaged mitochondria overproduce the superoxide anion, which escape the mitochondria to undergo a series of reactions to form mtROS. mtROS overproduction is sensed by TRX-interaction protein (TXNIP) or mitochondrial DNA (mtDNA), which bind to the leucine-rich repeat region of NLRP3 and lead to NLRP3 inflammasome activation. Consequently, the microglia is activated, which further promotes neuroinflammation and induces neuronal apoptosis, contributing to cognitive dysfunction.

The hypothesis we present here is that mtROS-induced NLRP3 activation may be a pivotal upstream mechanism that controls microglial IL-1β cleavage and secretion, and subsequent IL-1β-mediated inflammatory cascades in the hippocampus. Therefore, the mtROS/NLRP3 inflammasome/IL-1β signaling pathway may be a new drug target for attenuating POCD (Figure 1).

## Evaluation of the Hypothesis

### IL-1β-Mediated Neuroinflammation Following Surgery and POCD

The activation of microglia induced by surgery and the resulting exacerbated inflammatory response in the hippocampus have been associated with impaired cognitive function (Hovens et al., 2014). Tissue damage following surgery engages the immune system and produces a wide range of inflammatory cytokines and macrophages in the serum (Terrando et al., 2010, 2011). These cytokines, including IL-1β, IL-6, and high mobility group box-1 (HMGB-1), inhibit Wnt/β-catenin/Annexin A1 signaling pathway and disrupt the BBB integrity, which facilitates the migration of macrophages into areas of the brain, in particular, but not restricted to the hippocampus (Hu et al., 2016; Fan et al., 2018). Additionally, surgical stress and anesthetics promote deposition of oligomeric tau and Aβ paralleled by increased levels of ROS in cerebral microvasculature, which disrupts BBB integrity and shifts the balance of neuroimmune microenvironment toward proinflammatory milieu (Arora et al., 2015; Castillo-Carranza et al., 2017; Zou et al., 2018). Subsequently, the inhibition of anti-inflammatory receptors (e.g., nicotinic acetylcholine receptors; Liu P.-R. et al., 2017) and activation of pro-inflammatory receptors [e.g., toll-like receptor 4 (TLR 4)] trigger a cascade of downstream signaling events (Terrando et al., 2010). Consequently, microglia in the hippocampus is activated, which further promotes neuroinflammation and induces neuronal apoptosis, contributing to cognitive dysfunction (Terrando et al., 2011).

POCD involves a wide range of cognitive impairment and multiple brain regions are involved in cognitive processes of POCD, such as hippocampus, prefrontal cortex, striatum and amygdala (Hovens et al., 2015). However, the role of the hippocampus in many of the processes is particularly well established (Skvarc et al., 2018). Since inflammatory cytokine receptors are highly concentrated in areas associated with learning and memory (Parnet et al., 2002), particularly in the regions of the hippocampus, surgery-induced neuroinflammation in the brain may primarily disrupt hippocampus-dependent learning and memory (Zhang et al., 2016). The hippocampus arguably contains the largest number of receptors for IL-1β and is implicated in optimal memory and learning processes (Gemma et al., 2005). This region of the brain is also sensitive to the insults of aging (Huang et al., 2008) and excessive levels of IL-1β are associated with cognitive disorders in aging animal models (Barrientos et al., 2006; Chen et al., 2008). Therefore, we focused on the hippocampus in our hypothesis.

The hippocampal-dependent memory impairment was associated with IL-1β increase in the hippocampal structure of aged rats, while young rats did not present any exacerbated response to surgery and anesthesia (Barrientos et al., 2012). Hippocampal IL-1β-mediated neuroinflammation plays a pivotal role in surgery-induced cognitive impairment in aged rats (Goshen et al., 2007). A recent animal study reported that surgery induced significant morphological changes of microglial reactivity paralleled by elevations of IL-1β at 24 h and day 3 compared to naive and animals treated only with anesthesia, which showed that microglial reactivity after surgery may be the cause of increase in the levels of IL-1β in the hippocampus (Cibelli et al., 2010). Upon peripheral macrophage and resident microglia activation in the hippocampus, IL-1β is activated to enhance expression and release, triggering a cascade of downstream signaling events (Hovens et al., 2014; Skvarc et al., 2018). Elevated levels of IL-1β induce the subsequent production of IL-6 and HMGB-1, which further drive IL-1β expression, in a feed-forward mechanism promoting further activation of inflammatory signaling pathways (Lee et al., 2014). These cytokines have been described to result in impairment of hippocampal long-term potentiation, neuronal activity, and synaptic plasticity through modulation of glutamate receptors and inhibition of glutamate release in rats (Yirmiya and Goshen, 2011; Riazi et al., 2015).

The specificity of IL-1β involvement has been revealed by previous studies. A study conducted showed that a single intracisternal administration of IL-1 receptor antagonist (hIL-1RA; 112 μg) at the time of surgery was sufficient to block both the behavioral deficit and the neuroinflammatory response in the hippocampus of aged rats 4 day after surgery (Barrientos et al., 2012). Cibelli et al. (2010) further highlighted the importance of IL-1β expression in the neuroinflammatory effect of surgery and cognitive impairment. The results showed that reactive microgliosis in the hippocampus was no longer triggered following surgery in mice lacking the IL-1 receptor. These data reinforce the hypothesis that increased IL-1β levels in the hippocampus are likely to play a prominent role in the pathogenesis of POCD.

### NLRP3 Inflammasome Activation by mtROS in the Hippocampus and POCD

Evidence supports that IL-1β cleavage and secretion are primarily dependent on activation of the inflammasome, a multiprotein complex localized in the cytoplasm (Dempsey et al., 2017). Numerous inflammasomes, including NLRP1, NLRP3, and NLRC4, have been reported to exhibit inflammasome activity in several diseases (Schroder and Tschopp, 2010). Each NLR forms its own inflammasome and the NLRP3 inflammasome is only described as a central component in the production of IL-1β among them (Lamkanfi and Dixit, 2014). The NLRP3 inflammasome is a pattern recognition receptor and its activation depends on exposure to immune activators such as pathogen-associated molecular patterns, danger-associated molecular patterns, and environmental irritants (Shao et al., 2015). Immune activators cause a conformational change in NLRP3, which allows an interaction between the pyrin domains in NLRP3 and ASC (Cordero et al., 2018; Place and Kanneganti, 2018). Subsequently, ASC recruits pro-caspase-1 through its caspase recruitment domain, causing the activation of the NLRP3 inflammasome. The activated NLRP3 inflammasome triggers pro-caspase-1 self-cleavage and this complex releases cleaved caspase-1 into the cytosol, which induces the conversion of IL-1β from its immature “pro” forms to an active form, which is secreted (Willingham et al., 2009; Shao et al., 2015).

A non-canonical pathway downstream of caspase-1 is also involved in NLRP3-dependent IL-1β processing (Lamkanfi and Dixit, 2014). However, compared to the non-canonical pathway, canonical NLRP3 inflammasome controls systemic low grade age-related sterile inflammation in both periphery and the brain (Youm et al., 2013). Emerging evidence suggests that canonical NLRP3 inflammasome activation is linked to inflammation-mediated cognitive decline and neuropathological changes with aging. Reduction of canonical NLRP3 inflammasome-induced inflammation prevents aged-related cognitive dysfunction (Goldberg and Dixit, 2015; Wang et al., 2018). Studies on microglia and animal models have revealed an important role for the canonical NLRP3 inflammasome in AD pathogenesis (Heneka et al., 2013; Dempsey et al., 2017). Activation of the NLRP3 inflammasome contributes to Aβ accumulation, synaptic dysfunction, and cognitive impairment in APP/PS1 mice, suggesting that blocking the assembly of the inflammasome may constitute a novel therapeutic intervention for attenuating changes that negatively affect neuronal function in AD (Dempsey et al., 2017). Furthermore, preliminary experimental findings have suggested that NLRP3 inflammasome activation was linked to cognitive impairment after isoflurane anesthesia and isoflurane exposure induced the upregulation of NLRP3 and subsequently increased the level of IL-1β in the hippocampus of aged mice (Li et al., 2014; Wang et al., 2018). Despite a shortage of literature on sources of IL-1β in the pathophysiological mechanism of POCD, existing evidence shows that isoflurane-induced IL-1β overproduction in the hippocampus can be partially attenuated but not be repressed completely by an inhibitor of NLRP3-caspase-1 (Wang et al., 2018). Therefore, we hypothesize that canonical NLRP3 inflammasome/IL-1β axis is likely is implicated in postoperative inflammatory mediators-induced cognitive impairment.

Several theories have been proposed to explain the cellular signal responsible for activation of the NLRP3 inflammasome including cytosolic K^+^ efflux, the production of ROS, and the release of mtDNA and ROS (Lamkanfi and Dixit, 2014). The importance of K^+^ efflux in NLRP3 activation is supported by the fact that NLRP3 activators, such as ATP, nigericin and pore-forming toxins, result in lower intracellular concentration of K^+^, and a higher extracellular concentration of K^+^ inhibits activation of the NLRP3 inflammasome (He et al., 2016). The involvement of K^+^ efflux in NLRP3 inflammasome activation has been further suggested by a typical study showing that the drop in intracellular K^+^ concentration is the common step that is necessary and sufficient to engage the NLRP3 inflammasome activation (Munoz-Planillo et al., 2013). However, the mechanistic link between K^+^ efflux-induced NLRP3 activation and inflammation-mediated cognitive dysfunction remains poorly understood.

Alternative models for NLRP3 activation involve ROS production. Recent evidence suggests that ROS are positively correlated with cognitive impairment after surgery (Qiu et al., 2016b). ROS overproduction is an important upstream event that can activate NLRP3 inflammation and amplify the production of IL-1β (Qiu et al., 2016a). The source of ROS is currently unclear. The most studied ROS include nicotinamide adenine dinucleotide phosphate oxidase and mtROS (Circu and Aw, 2010), but mitochondria are considered to be the major source of intracellular ROS (Qiu et al., 2016b). Mitochondrial damage is a key mechanism of neurodegenerative disorders (Gao et al., 2017; Liu K. et al., 2017). Evidence from cellular and animal models indicates that exposure to anesthetics or surgical stress induces deficiencies in mitochondrial respiratory chain components and mtDNA mutations which could result in membrane potential loss and the opening of the mitochondrial permeability transition pore, causing increased leakage of electrons (Zhang et al., 2012; Li et al., 2017). Leaked electrons react with molecular oxygen to produce the superoxide anion, which escape the mitochondria to undergo a series of reactions to form mtROS (Kim et al., 2015). Iron-dependent mtROS is also tightly associated with neurodegenerative diseases (Gao et al., 2017). Increased iron accumulation and oxidative stress in the brain, especially in the hippocampus, may be involved in the pathogenesis of POCD (An et al., 2013). mtROS leads to accumulation of mtDNA mutations, increased superoxide production, and a vicious cycle of oxidative stress, which further accelerates mtDNA mutagenesis and damages mitochondrial function (Xu et al., 2017). Although the role of mtROS in NLRP3 activation is a topic of longstanding controversy, mounting studies have suggested that mtROS production may be linked to NLRP3 inflammasome activation (Schroder and Tschopp, 2010; Choi and Ryter, 2014; Shao et al., 2015; He et al., 2016). A study conducted by Wu et al. (2015) also showed that mtROS blockade by mitochondrion-targeted antioxidant SS-31 suppressed NLRP3 inflammasome activation and alleviated isoflurane-induced cognitive deficits 24 h after anesthesia. Since ROS are short-lived and act as a messenger only for a short distance, NLRP3 is thought to be localized in close proximity to mitochondria, which allows efficient sensing of the presence of ROS produced in the same cell by malfunctioning mitochondria (Zhou et al., 2011). Mechanisms directing mtROS-dependent NLRP3 inflammasome activation have been characterized in detail (Zhou et al., 2011; Alfonso-Loeches et al., 2014; Minutoli et al., 2016). mtROS overproduction is sensed by a complex of thioredoxin (TRX) and TRX-interaction protein (TXNIP) that induce the dissociation of the complex. Subsequently, TXNIP binds to the leucine-rich repeat region of NLRP3, leading to NLRP3 inflammasome activation (Minutoli et al., 2016). Another potential mechanism of mtROS and NLRP3 inflammasome activation is the release of mt DNA. mtDNA preceding mtROS production, escape from the mitochondria to the cytoplasm *via* mitochondrial membrane permeability transition pores and mtDNA directly binds and activates the NLRP3 inflammasome (Kim et al., 2015). While the exact pathway by which mtROS mediates NLRP3 inflammasome activation and assembly remains elusive, these existing findings suggest that mitochondrial dysfunction and mtROS overexpression may at least be partly responsible for expression of cytokines *via* NLRP3 inflammasome activation in POCD.

### The Cholinergic Modulation of mtROS/NLRP3 Inflammasome/IL-1β Signaling Pathway

Cholinergic transmission plays a key role in cognitive function. A cerebral cholinergic deficit has been demonstrated to be implicated in age-related cognitive impairment (Rossi et al., 2014). Activation of the cholinergic anti-inflammatory pathway suppresses excessive neuroinflammation in neurodegenerative diseases, such as POCD, AD, multiple sclerosis and Parkinson’s disease (Taly et al., 2009; Leslie, 2017). The α7 nicotinic acetylcholine receptor (α7nAChR) is identified as an essential mediator of cholinergic anti-inflammatory pathway (Pavlov and Tracey, 2017). The dysfunctional activity of α7nAchR in the hippocampus in response to surgical stress and anesthesia is now considered to be the critical event in the development of neuroinflammation in aged POCD rats (Liu P.-R. et al., 2017). Recently, we and other research groups have reported that α7nAChR agonists resolved IL-1β-mediated neuroinflammation and reversed cognitive decline after surgery in animal studies (Chen et al., 2018; Wei et al., 2018). Therefore, we speculate that cholinergic deficit induces neuroinflammation in POCD and α7nAChR may be the potential upstream of mtROS/NLRP3 inflammasome/IL-1β signaling pathway in microglia.

Activation of α7nAChR suppresses the NLRP3 inflammasome, but the nature of this suppression is unclear (Hecker et al., 2015; Ke et al., 2017). One potential mechanism involves the autophagic removal of mtROS production. Autophagy is a cellular quality-control system which removes unnecessary or damaged proteins and organelles *via* the lysosomal apparatus (Zhang et al., 2018). Autophagy has been demonstrated to regulate inflammation and immune responses in the Central Nervous System, especially in inflammatory cells such as microglia and astrocyte (De Luca et al., 2018). Deficient autophagy impairs mitochondrial integrity and promotes generation of mtROS, consequently contributing to activation of inflammasome (Razani et al., 2012). Defects in autophagy, along with neuroinflammation, have been implicated in the pathogenesis of postoperative cognitive decline (von Haefen et al., 2018). Additionally, evidence has shown that activating α7nAChR enhances microglial autophagy, which suppresses neuroinflammation and thus plays an alleviative role in neurodegenerative disorders (Jeong and Park, 2015; Shao et al., 2017). Collectively, these findings support the hypothesis that autophagy deficiency induced by dysfunctional α7nAChR promotes POCD *via* the activation of mtROS/NLRP3 inflammasome/IL-1β signaling pathway in microglia.

### Other Potential Signaling Pathways Involved in Modulating of Neuroinflammation in POCD

The mechanism initiating, controlling and modulating neuroinflammation in POCD are complex. The non-canonical inflammasomes/caspase-11 is also required for macrophages to secrete IL-1β (Lamkanfi and Dixit, 2014). The finding highlights the potential benefits of blocking the caspase-11 directly over inhibiting IL-1β expression and its downstream cytokines. Recent studies have identified additional signaling pathways that can regulate cytokine synthesis and release in the pathogenesis of POCD, such as TNF-α/TLR4 (Terrando et al., 2010), ATP/P2X7 (Zheng et al., 2017) and cannabinoid receptor type 2 (CB2R)-related signaling pathways (Sun et al., 2017). Additionally, certain key signaling pathways/molecules associated with the regulation of inflammation, such as AMPK, mTOR, Nur77, and miRNAs, have been demonstrated to play critical roles in chronic age-related diseases (Wei et al., 2016; Xu et al., 2017; Li et al., 2018; Zhang et al., 2018). However, the involvement of these signaling pathways/molecules in POCD remains to be explored.

## Important Implications of The Hypothesis and Proposed Experimental Plan

Although numerous drugs with neuroprotective action during surgery and anesthesia have been studied, there is no agreement on the efficiency of prophylactic neuroprotectants in POCD. If our hypothesis is verified by future studies, the NLRP3 inflammasome will become a new drug target for attenuating POCD.

Several small molecules including MCC950 (Coll et al., 2015; Dempsey et al., 2017) and BHB (Youm et al., 2015) have been shown to specifically inhibit the NLRP3 inflammasome activation. Other types of NLRP3 inflammasome inhibitors, such as autophagy inducer (Resveratrol, arglabin and CB2R agonist), type I interferon (IFN) and IFN-β (Malhotra et al., 2015) and microRNA223, have also been reported (Shao et al., 2015), although these agents have limited potency and are non-specific. The most promising inhibitor of NLRP3 inflammasome activation was described in a groundbreaking report in *Nature Medicine* in 2015 (Coll et al., 2015). Coll et al. (2015) showed that MCC950 inhibited canonical and non-canonical NLRP3 activation at nanomolar concentrations in mouse bone marrow-derived macrophages (BMDMs) and the half-maximal inhibitory concentration of MCC950 was approximately 7.5 nM in BMDMs. The study further showed that MCC950 specifically inhibited NLRP3 inflammasome but not NLRP1 or NLRC4 activation. MCC950 has been demonstrated to present certain advantages over other inhibitors of the NLRP3 inflammasome. Compared to other inhibitors, MCC950 may have less immunosuppressive effects because it specifically targets NLRP3 and does not result in the complete blockade of IL-1β which is essential during infection and antimicrobial responses, especially for elderly and immunosuppressed populations (Lopez-Castejon and Pelegrin, 2012; Coll et al., 2015). As a small molecule, numerous studies have shown that MCC950 effectively traversed an impaired BBB and attenuated neuroinflammation-related diseases. Dempsey et al. (2017) showed that MCC950-treated APP/PS1 mice performed significantly better than control-treated APP/PS1 mice, and MCC950 promoted non-phlogistic clearance of Aβ_1–40_ and Aβ_1–42_ in APP/PS1 mice. They further showed that the impact of MCC950 on Aβ pathology resulted from its ability to block production of IL-1β while it promoted Aβ phagocytosis by microglia. MCC950 has also been demonstrated to reduce the neurological deficit score of 24 h after cerebral ischemia reperfusion and improved the 28-day survival rate of cerebral ischemia-reperfusion injury in diabetic mice (Hong et al., 2018). Another study has further revealed that MCC950 reduced neuroinflammation and improved the long-term neurological outcomes on the 3, 7, and 14 days after traumatic brain injury in a murine model and the therapeutic window for MCC950 against traumatic brain injury was as long as 6 h (Xu et al., 2018). BHB, produced in the liver, is another promising small molecule inhibitor of NLRP3 inflammasome. BHB serves as an alternative energy source for the brain, heart, and skeletal muscle in mammals during states of energy deficit (Newman and Verdin, 2014). Like MCC950, BHB specifically suppresses activation of the NLRP3 inflammasome, without affecting NLRC4, AIM2 or non-canonical caspase-11 inflammasome activation (Youm et al., 2015). However, their mechanisms differ in key aspects. Youm et al. (2015) discovered that BHB inhibited the NLRP3 inflammasome by preventing K^+^ efflux and reducing ASC oligomerization and speck formation. Furthermore, a recent study has shown that BHB attenuated stress-induced behavioral as well as the elevation of IL-1β and TNF-α in the rodent hippocampus by inhibiting NLRP3 inflammasome activation (Yamanashi et al., 2017).

Literature pertaining to clinical trials associated with NLRP3 inflammasome and POCD is scarce. Thus, a basic hypothesis involving microglial cells, mice hippocampi and behavioral studies is provided here. Experimentally, it proposes the use of animal and cellular models of POCD to investigate the following: (i) NLRP3 inflammasome mediated IL-1β activation and hippocampus-dependent cognitive performance; (ii) mitochondrial oxidative stress and NLRP3 inflammasome activation in the hippocampus and microglia; and (iii) mtROS and its regulatory effect on the NLRP3 inflammasome. To evaluate whether NLRP3 inflammasome mediated IL-1β activation is involved in POCD, NLRP3^−/−^ or caspase-1^−/−^ aged mice carrying mutations (e.g., C57/Bl6 background) and primary microglia prepared from neonatal mice are recommended in future studies (Heneka et al., 2013). Surgery is performed under general anesthesia and the primary microglia are preincubated with LPS and isoflurane (Wang et al., 2018). The Morris Water Maze test should be performed to evaluate the hippocampus-dependent spatial learning and memory, and the assembly and interaction of the complex consisting of NLRP3, ASC, and caspase-1 in microglia should be tested using a series of immunological and biochemical assays, such as small interference ribonucleic acid, immunohistochemistry, and co-immunoprecipitation (Dempsey et al., 2017; Wang et al., 2018). To understand whether mtROS and its regulatory effect on the NLRP3 inflammasome are involved in POCD, flow cytometry and confocal microscopy should be carried out in primary microglia or hippocampus after exposure to the inhibitors or enhancers of NLRP3 inflammasome (e.g., MCC950 and BHB) and mtROS (e.g., Rotenone and SS-31; Zhou et al., 2011).

Future clinical trials with MCC950 and BHB may contribute to the development of new anti-inflammatory therapies for neuroinflammation-associated diseases. Therefore, we hypothesize that the two small molecule inhibitors, especially MCC950, may be viable for the attenuation of patients with POCD in the future.

Additionally, α7nAChR and autophagy may be potential drug targets for attenuating POCD by directly regulating mtROS/NLRP3 inflammasome/IL-1β signaling pathway. Terrando et al. (2011) have highlighted the importance of α7nAChR in resolving the inflammatory pathogenesis of POCD. α7nAChR agonists prevent macrophage migration into the hippocampus and cognitive decline following surgery. A recent research has also shown that activated α7nAChR markedly improved cognitive impairment after cardiopulmonary bypass in rats (Chen et al., 2018). Pre-clinical evidence suggested that inducing autophagy was effective in protecting against several neurodegenerative diseases, though this is not a universal finding (Zou et al., 2017; Weng et al., 2018). Recently, it has been reported that enhancement of autophagy could ameliorate the pathogenesis of cognitive impairment in aged hippocampus after propofol anesthesia (Yang et al., 2017). Activating α7nAChR and inducing autophagy might also provide a potential therapeutic target for POCD.

## Conclusions

In conclusion, mitochondrial dysfunction in POCD triggers mtROS generation and an mtROS-dependent pathway may be responsible for NLRP3 inflammasome complex formation in the hippocampus, which may be regulated by α7nAChR and autophagy. Subsequently, pro-caspase-1 clustering induces autoactivation and caspase-1-dependent maturation and secretion of IL-1β. IL-1β further drives neuroinflammation in a feed-forward mechanism, which promotes subsequent activation of inflammatory cytokines and eventually causes neuronal apoptosis (Figure 1). The mtROS/NLRP3 inflammasome/IL-1β signaling pathway may be a potential drug target for therapeutic intervention in POCD.

## Author Contributions

PW, FY, and JL wrote the draft. PW, QZ, and WT created the figure. JL secured the funds to support this project. All the authors read and approved the manuscript.

## Conflict of Interest Statement

The authors declare that the research was conducted in the absence of any commercial or financial relationships that could be construed as a potential conflict of interest.

## References

[B1] Alfonso-Loeches S. Urena-Peralta J. R. Morillo-Bargues M. J. Oliver-De La Cruz J. Guerri C. (2014). Role of mitochondria ROS generation in ethanol-induced NLRP3 inflammasome activation and cell death in astroglial cells. Front. Cell. Neurosci. 8:216. 10.3389/fncel.2014.00216 25136295 PMC4118026

[B2] An L. N. Yue Y. Guo W. Z. Miao Y. L. Mi W. D. Zhang H.. (2013). Surgical trauma induces iron accumulation and oxidative stress in a rodent model of postoperative cognitive dysfunction. Biol. Trace Elem. Res. 151, 277–283. 10.1007/s12011-012-9564-9 23229539

[B3] Arora K. Cheng J. Nichols R. A. (2015). Nicotinic acetylcholine receptors sensitize a MAPK-linked toxicity pathway on prolonged exposure to β-amyloid. J. Biol. Chem. 290, 21409–21420. 10.1074/jbc.m114.634162 26139609 PMC4571869

[B4] Barrientos R. M. Hein A. M. Frank M. G. Watkins L. R. Maier S. F. (2012). Intracisternal interleukin-1 receptor antagonist prevents postoperative cognitive decline and neuroinflammatory response in aged rats. J. Neurosci. 32, 14641–14648. 10.1523/JNEUROSCI.2173-12.2012 23077050 PMC3492959

[B5] Barrientos R. M. Higgins E. A. Biedenkapp J. C. Sprunger D. B. Wright-Hardesty K. J. Watkins L. R.. (2006). Peripheral infection and aging interact to impair hippocampal memory consolidation. Neurobiol. Aging 27, 723–732. 10.1016/j.neurobiolaging.2005.03.010 15893410

[B6] Castillo-Carranza D. L. Nilson A. N. Van Skike C. E. Jahrling J. B. Patel K. Garach P.. (2017). Cerebral microvascular accumulation of tau oligomers in Alzheimer’s disease and related tauopathies. Aging Dis. 8, 257–266. 10.14336/AD.2017.0112 28580182 PMC5440106

[B7] Chen J. Buchanan J. B. Sparkman N. L. Godbout J. P. Freund G. G. Johnson R. W. (2008). Neuroinflammation and disruption in working memory in aged mice after acute stimulation of the peripheral innate immune system. Brain Behav. Immun. 22, 301–311. 10.1016/j.bbi.2007.08.014 17951027 PMC2374919

[B8] Chen K. Sun Y. Dong W. Zhang T. Zhou N. Yu W.. (2018). Activated alpha7nachr improves postoperative cognitive dysfunction and intestinal injury induced by cardiopulmonary bypass in rats: inhibition of the proinflammatory response through the th17 immune response. Cell. Physiol. Biochem. 46, 1175–1188. 10.1159/000489068 29672286

[B9] Choi A. J. Ryter S. W. (2014). Inflammasomes: molecular regulation and implications for metabolic and cognitive diseases. Mol. Cells 37, 441–448. 10.14348/molcells.2014.0104 24850149 PMC4086337

[B10] Cibelli M. Fidalgo A. R. Terrando N. Ma D. Monaco C. Feldmann M.. (2010). Role of interleukin-1beta in postoperative cognitive dysfunction. Ann. Neurol. 68, 360–368. 10.1002/ana.22082 20818791 PMC4836445

[B11] Circu M. L. Aw T. Y. (2010). Reactive oxygen species, cellular redox systems and apoptosis. Free Radic. Biol. Med. 48, 749–762. 10.1016/j.freeradbiomed.2009.12.022 20045723 PMC2823977

[B12] Coll R. C. Robertson A. A. Chae J. J. Higgins S. C. Munoz-Planillo R. Inserra M. C.. (2015). A small-molecule inhibitor of the NLRP3 inflammasome for the treatment of inflammatory diseases. Nat. Med. 21, 248–255. 10.1038/nm.3806 25686105 PMC4392179

[B13] Cordero M. D. Williams M. R. Ryffel B. (2018). AMP-activated protein kinase regulation of the NLRP3 inflammasome during aging. Trends Endocrinol. Metab. 29, 8–17. 10.1016/j.tem.2017.10.009 29150317

[B14] De Luca C. Colangelo A. M. Alberghina L. Papa M. (2018). Neuro-immune hemostasis: homeostasis and diseases in the central nervous system. Front. Cell. Neurosci. 12:459. 10.3389/fncel.2018.00459 30534057 PMC6275309

[B15] Dempsey C. Rubio Araiz A. Bryson K. J. Finucane O. Larkin C. Mills E. L.. (2017). Inhibiting the NLRP3 inflammasome with MCC950 promotes non-phlogistic clearance of amyloid-beta and cognitive function in APP/PS1 mice. Brain Behav. Immun. 61, 306–316. 10.1016/j.bbi.2016.12.014 28003153

[B16] Evered L. Scott D. A. Silbert B. Maruff P. (2011). Postoperative cognitive dysfunction is independent of type of surgery and anesthetic. Anesth. Analg. 112, 1179–1185. 10.1213/ANE.0b013e318215217e 21474666

[B17] Fan J. An X. Yang Y. Xu H. Fan L. Deng L.. (2018). MiR-1292 targets FZD4 to regulate senescence and osteogenic differentiation of stem cells in TE/SJ/Mesenchymal tissue system via the wnt/beta-catenin pathway. Aging Dis. 9, 1103–1121. 10.14336/AD.2018.1110 30574422 PMC6284756

[B18] Gao G. Zhang N. Wang Y. Q. Wu Q. Yu P. Shi Z. H.. (2017). Mitochondrial ferritin protects hydrogen peroxide-induced neuronal cell damage. Aging Dis. 8, 458–470. 10.14336/ad.2016.1108 28840060 PMC5524808

[B19] Gemma C. Fister M. Hudson C. Bickford P. C. (2005). Improvement of memory for context by inhibition of caspase-1 in aged rats. Eur. J. Neurosci. 22, 1751–1756. 10.1111/j.1460-9568.2005.04334.x 16197515

[B20] Goldberg E. L. Dixit V. D. (2015). Drivers of age-related inflammation and strategies for healthspan extension. Immunol. Rev. 265, 63–74. 10.1111/imr.12295 25879284 PMC4400872

[B21] Goshen I. Kreisel T. Ounallah-Saad H. Renbaum P. Zalzstein Y. Ben-Hur T.. (2007). A dual role for interleukin-1 in hippocampal-dependent memory processes. Psychoneuroendocrinology 32, 1106–1115. 10.1016/j.psyneuen.2007.09.004 17976923

[B22] He Y. Hara H. Nunez G. (2016). Mechanism and regulation of NLRP3 inflammasome activation. Trends Biochem. Sci. 41, 1012–1021. 10.1016/j.tibs.2016.09.002 27669650 PMC5123939

[B23] Hecker A. Kullmar M. Wilker S. Richter K. Zakrzewicz A. Atanasova S.. (2015). Phosphocholine-modified macromolecules and canonical nicotinic agonists inhibit ATP-induced IL-1beta release. J. Immunol. 195, 2325–2334. 10.4049/jimmunol.1400974 26202987

[B24] Heneka M. T. Kummer M. P. Stutz A. Delekate A. Schwartz S. Vieira-Saecker A.. (2013). NLRP3 is activated in Alzheimer’s disease and contributes to pathology in APP/PS1 mice. Nature 493, 674–678. 10.1038/nature11729 23254930 PMC3812809

[B25] Hong P. Li F. X. Gu R. N. Fang Y. Y. Lai L. Y. Wang Y. W.. (2018). Inhibition of NLRP3 inflammasome ameliorates cerebral ischemia-reperfusion injury in diabetic mice. Neural Plast. 2018:9163521. 10.1155/2018/9163521 29853850 PMC5941718

[B26] Hovens I. B. Schoemaker R. G. van der Zee E. A. Absalom A. R. Heineman E. van Leeuwen B. L. (2014). Postoperative cognitive dysfunction: involvement of neuroinflammation and neuronal functioning. Brain Behav. Immun. 38, 202–210. 10.1016/j.bbi.2014.02.002 24517920

[B27] Hovens I. B. van Leeuwen B. L. Nyakas C. Heineman E. van der Zee E. A. Schoemaker R. G. (2015). Postoperative cognitive dysfunction and microglial activation in associated brain regions in old rats. Neurobiol. Learn. Mem. 118, 74–79. 10.1016/j.nlm.2014.11.009 25460037

[B28] Hu N. Wang C. Zheng Y. Ao J. Zhang C. Xie K.. (2016). The role of the Wnt/beta-catenin-Annexin A1 pathway in the process of sevoflurane-induced cognitive dysfunction. J. Neurochem. 137, 240–252. 10.1111/jnc.13569 26851642

[B29] Huang Y. Henry C. J. Dantzer R. Johnson R. W. Godbout J. P. (2008). Exaggerated sickness behavior and brain proinflammatory cytokine expression in aged mice in response to intracerebroventricular lipopolysaccharide. Neurobiol. Aging 29, 1744–1753. 10.1016/j.neurobiolaging.2007.04.012 17543422 PMC2647751

[B30] Jeong J. K. Park S. Y. (2015). Melatonin regulates the autophagic flux via activation of alpha-7 nicotinic acetylcholine receptors. J. Pineal. Res. 59, 24–37. 10.1111/jpi.12235 25808024

[B31] Ke P. Shao B. Z. Xu Z. Q. Chen X. W. Wei W. Liu C. (2017). Activating alpha7 nicotinic acetylcholine receptor inhibits NLRP3 inflammasome through regulation of beta-arrestin-1. CNS Neurosci. Ther. 23, 875–884. 10.1111/cns.12758 28941191 PMC6492746

[B32] Kim H. K. Chen W. Andreazza A. C. (2015). The potential role of the NLRP3 inflammasome as a link between mitochondrial complex I dysfunction and inflammation in bipolar disorder. Neural Plast. 2015:408136. 10.1155/2015/408136 26075098 PMC4444590

[B33] Lamkanfi M. Dixit V. M. (2014). Mechanisms and functions of inflammasomes. Cell 157, 1013–1022. 10.1016/j.cell.2014.04.007 24855941

[B34] Lee Y. H. Su S. B. Huang C. C. Sheu H. M. Tsai J. C. Lin C. H.. (2014). N-acetylcysteine attenuates hexavalent chromium-induced hypersensitivity through inhibition of cell death, ROS-related signaling and cytokine expression. PLoS One 9:e108317. 10.1371/journal.pone.0108317 25248126 PMC4172727

[B35] Leslie M. (2017). The post-op brain. Science 356, 898–900. 10.1126/science.356.6341.898 28572347

[B36] Li H. Fan J. Fan L. Li T. Yang Y. Xu H.. (2018). MiRNA-10b reciprocally stimulates osteogenesis and inhibits adipogenesis partly through the TGF-beta/SMAD2 signaling pathway. Aging Dis. 9, 1058–1073. 10.14336/ad.2018.0214 30574418 PMC6284771

[B37] Li X. M. Zhou M. T. Wang X. M. Ji M. H. Zhou Z. Q. Yang J. J. (2014). Resveratrol pretreatment attenuates the isoflurane-induced cognitive impairment through its anti-inflammation and -apoptosis actions in aged mice. J. Mol. Neurosci. 52, 286–293. 10.1007/s12031-013-0141-2 24126892

[B38] Li Z. Ni C. Xia C. Jaw J. Wang Y. Cao Y.. (2017). Calcineurin/nuclear factor-kappaB signaling mediates isoflurane-induced hippocampal neuroinflammation and subsequent cognitive impairment in aged rats. Mol. Med. Rep. 15, 201–209. 10.3892/mmr.2016.5967 27909728 PMC5355741

[B39] Liu K. Zang Y. Guo X. Wei F. Yin J. Pang L.. (2017). The Δ133p53 isoform reduces Wtp53-induced stimulation of DNA Pol γ activity in the presence and absence of D4T. Aging Dis 8, 228–239. 10.14336/AD.2016.0910 28400988 PMC5362181

[B40] Liu P.-R. Zhou Y. Zhang Y. Diao S. (2017). Electroacupuncture alleviates surgery-induced cognitive dysfunction by increasing α7-nAChR expression and inhibiting inflammatory pathway in aged rats. Neurosci. Lett. 659, 1–6. 10.1016/j.neulet.2017.08.043 28842280

[B41] Lopez-Castejon G. Pelegrin P. (2012). Current status of inflammasome blockers as anti-inflammatory drugs. Expert Opin. Investig. Drugs 21, 995–1007. 10.1517/13543784.2012.690032 22612568

[B42] Malhotra S. Rio J. Urcelay E. Nurtdinov R. Bustamante M. F. Fernandez O.. (2015). NLRP3 inflammasome is associated with the response to IFN-beta in patients with multiple sclerosis. Brain 138, 644–652. 10.1093/brain/awu388 25586466 PMC4408432

[B43] Minutoli L. Puzzolo D. Rinaldi M. Irrera N. Marini H. Arcoraci V.. (2016). ROS-mediated NLRP3 inflammasome activation in brain, heart, kidney and testis ischemia/reperfusion injury. Oxid. Med. Cell. Longev. 2016:2183026. 10.1155/2016/2183026 27127546 PMC4835650

[B44] Munoz-Planillo R. Kuffa P. Martinez-Colon G. Smith B. L. Rajendiran T. M. Nunez G. (2013). K^+^ efflux is the common trigger of NLRP3 inflammasome activation by bacterial toxins and particulate matter. Immunity 38, 1142–1153. 10.1016/j.immuni.2013.05.016 23809161 PMC3730833

[B45] Newman J. C. Verdin E. (2014). Ketone bodies as signaling metabolites. Trends Endocrinol. Metab. 25, 42–52. 10.1016/j.tem.2013.09.002 24140022 PMC4176946

[B46] Parnet P. Kelley K. W. Bluthe R. M. Dantzer R. (2002). Expression and regulation of interleukin-1 receptors in the brain. Role in cytokines-induced sickness behavior. J. Neuroimmunol. 125, 5–14. 10.1016/s0165-5728(02)00022-x 11960635

[B47] Pavlov V. A. Tracey K. J. (2017). Neural regulation of immunity: molecular mechanisms and clinical translation. Nat. Neurosci. 20, 156–166. 10.1038/nn.4477 28092663

[B48] Place D. E. Kanneganti T. D. (2018). Recent advances in inflammasome biology. Curr. Opin. Immunol. 50, 32–38. 10.1016/j.coi.2017.10.011 29128729 PMC5857399

[B49] Qiu L. L. Ji M. H. Zhang H. Yang J. J. Sun X. R. Tang H.. (2016a). NADPH oxidase 2-derived reactive oxygen species in the hippocampus might contribute to microglial activation in postoperative cognitive dysfunction in aged mice. Brain Behav. Immun. 51, 109–118. 10.1016/j.bbi.2015.08.002 26254234

[B50] Qiu L. L. Luo D. Zhang H. Shi Y. S. Li Y. J. Wu D.. (2016b). Nox-2-mediated phenotype loss of hippocampal parvalbumin interneurons might contribute to postoperative cognitive decline in aging mice. Front. Aging Neurosci. 8:234. 10.3389/fnagi.2016.00234 27790135 PMC5062642

[B51] Razani B. Feng C. Coleman T. Emanuel R. Wen H. Hwang S.. (2012). Autophagy links inflammasomes to atherosclerotic progression. Cell Metab. 15, 534–544. 10.1016/j.cmet.2012.02.011 22440612 PMC3322320

[B52] Riazi K. Galic M. A. Kentner A. C. Reid A. Y. Sharkey K. A. Pittman Q. J. (2015). Microglia-dependent alteration of glutamatergic synaptic transmission and plasticity in the hippocampus during peripheral inflammation. J. Neurosci. 35, 4942–4952. 10.1523/JNEUROSCI.4485-14.2015 25810524 PMC6705378

[B53] Rossi A. Burkhart C. Dell-Kuster S. Pollock B. G. Strebel S. P. Monsch A. U.. (2014). Serum anticholinergic activity and postoperative cognitive dysfunction in elderly patients. Anesth. Analg. 119, 947–955. 10.1213/ANE.0000000000000390 25089730

[B54] Schroder K. Tschopp J. (2010). The inflammasomes. Cell 140, 821–832. 10.1016/j.cell.2010.01.040 20303873

[B55] Shao B.-Z. Ke P. Xu Z.-Q. Wei W. Cheng M.-H. Han B.-Z.. (2017). Autophagy plays an important role in anti-inflammatory mechanisms stimulated by alpha7 nicotinic acetylcholine receptor. Front. Immunol. 8:553. 10.3389/fimmu.2017.00553 28559895 PMC5432615

[B56] Shao B. Z. Xu Z. Q. Han B. Z. Su D. F. Liu C. (2015). NLRP3 inflammasome and its inhibitors: a review. Front. Pharmacol. 6:262. 10.3389/fphar.2015.00262 26594174 PMC4633676

[B57] Shoair O. A. Grasso Ii M. P. Lahaye L. A. Daniel R. Biddle C. J. Slattum P. W. (2015). Incidence and risk factors for postoperative cognitive dysfunction in older adults undergoing major noncardiac surgery: A prospective study. J. Anaesthesiol. Clin. Pharmacol. 31, 30–36. 10.4103/0970-9185.150530 25788770 PMC4353149

[B58] Skvarc D. R. Berk M. Byrne L. K. Dean O. M. Dodd S. Lewis M.. (2018). Post-operative cognitive dysfunction: an exploration of the inflammatory hypothesis and novel therapies. Neurosci. Biobehav. Rev. 84, 116–133. 10.1016/j.neubiorev.2017.11.011 29180259

[B59] Steinmetz J. Christensen K. B. Lund T. Lohse N. Rasmussen L. S. (2009). Long-term consequences of postoperative cognitive dysfunction. Anesthesiology 110, 548–555. 10.1097/ALN.0b013e318195b569 19225398

[B60] Sun L. Dong R. Xu X. Yang X. Peng M. (2017). Activation of cannabinoid receptor type 2 attenuates surgery-induced cognitive impairment in mice through anti-inflammatory activity. J. Neuroinflamm. 14:138. 10.1186/s12974-017-0913-7 28724382 PMC5518095

[B61] Taly A. Corringer P. J. Guedin D. Lestage P. Changeux J. P. (2009). Nicotinic receptors: allosteric transitions and therapeutic targets in the nervous system. Nat. Rev. Drug Discov. 8, 733–750. 10.1038/nrd2927 19721446

[B62] Terrando N. Eriksson L. I. Ryu J. K. Yang T. Monaco C. Feldmann M.. (2011). Resolving postoperative neuroinflammation and cognitive decline. Ann. Neurol. 70, 986–995. 10.1002/ana.22664 22190370 PMC4556354

[B63] Terrando N. Monaco C. Ma D. Foxwell B. M. Feldmann M. Maze M. (2010). Tumor necrosis factor-alpha triggers a cytokine cascade yielding postoperative cognitive decline. Proc. Natl. Acad. Sci. U S A 107, 20518–20522. 10.1073/pnas.1014557107 21041647 PMC2996666

[B64] von Haefen C. Sifringer M. Endesfelder S. Kalb A. Gonzalez-Lopez A. Tegethoff A.. (2018). Physostigmine restores impaired autophagy in the rat hippocampus after surgery stress and LPS treatment. J. Neuroimmune. Pharmacol. 13, 383–395. 10.1007/s11481-018-9790-9 29790105

[B65] Wang W. Wang Y. Wu H. Lei L. Xu S. Shen X.. (2014). Postoperative cognitive dysfunction: current developments in mechanism and prevention. Med. Sci. Monit. 20, 1908–1912. 10.12659/MSM.892485 25306127 PMC4206478

[B66] Wang Z. Meng S. Cao L. Chen Y. Zuo Z. Peng S. (2018). Critical role of NLRP3-caspase-1 pathway in age-dependent isoflurane-induced microglial inflammatory response and cognitive impairment. J. Neuroinflammation 15:109. 10.1186/s12974-018-1137-1 29665808 PMC5904978

[B67] Wei P. Zheng Q. Liu H. Wan T. Zhou J. Li D.. (2018). Nicotine-induced neuroprotection against cognitive dysfunction after partial hepatectomy involves activation of BDNF/TrkB signaling pathway and inhibition of NF-kappaB signaling pathway in aged rats. Nicotine. Tob. Res. 20, 515–522. 10.1093/ntr/ntx157 29065194

[B68] Wei X. Gao H. Zou J. Liu X. Chen D. Liao J.. (2016). Contra-directional coupling of nur77 and nurr1 in neurodegeneration: a novel mechanism for memantine-induced anti-inflammation and anti-mitochondrial impairment. Mol. Neurobiol. 53, 5876–5892. 10.1007/s12035-015-9477-7 26497037

[B69] Weng R. Wei X. Yu B. Zhu S. Yang X. Xie F.. (2018). Combined measurement of plasma cystatin C and low-density lipoprotein cholesterol: A valuable tool for evaluating progressive supranuclear palsy. Parkinsonism Relat. Disord 52, 37–42. 10.1016/j.parkreldis.2018.03.014 29574085

[B70] Willingham S. B. Allen I. C. Bergstralh D. T. Brickey W. J. Huang M. T. Taxman D. J.. (2009). NLRP3 (NALP3, Cryopyrin) facilitates in vivo caspase-1 activation, necrosis and HMGB1 release via inflammasome-dependent and -independent pathways. J. Immunol. 183, 2008–2015. 10.4049/jimmunol.0900138 19587006 PMC3652593

[B71] Wu J. Li H. Sun X. Zhang H. Hao S. Ji M.. (2015). A mitochondrion-targeted antioxidant ameliorates isoflurane-induced cognitive deficits in aging mice. PLoS One 10:e0138256. 10.1371/journal.pone.0138256 26379247 PMC4575031

[B72] Xie F. Gao X. Yang W. Chang Z. Yang X. Wei X.. (2018). Advances in the research of risk factors and prodromal biomarkers of Parkinson’s disease. ACS Chem. Neurosci. [Epub ahead of print]. 10.1007/978-0-387-72076-0_6 30590011

[B73] Xu X. Yin D. Ren H. Gao W. Li F. Sun D.. (2018). Selective NLRP3 inflammasome inhibitor reduces neuroinflammation and improves long-term neurological outcomes in a murine model of traumatic brain injury. Neurobiol. Dis. 117, 15–27. 10.1016/j.nbd.2018.05.016 29859317

[B74] Xu Z. Feng W. Shen Q. Yu N. Yu K. Wang S.. (2017). Rhizoma coptidis and berberine as a natural drug to combat aging and aging-related diseases via anti-oxidation and AMPK activation. Aging Dis. 8, 760–777. 10.14336/AD.2016.0620 29344415 PMC5758350

[B75] Yamanashi T. Iwata M. Kamiya N. Tsunetomi K. Kajitani N. Wada N.. (2017). Beta-hydroxybutyrate, an endogenic NLRP3 inflammasome inhibitor, attenuates stress-induced behavioral and inflammatory responses. Sci. Rep. 7:7677. 10.1038/s41598-017-08055-1 28794421 PMC5550422

[B76] Yang N. Li L. Li Z. Ni C. Cao Y. Liu T.. (2017). Protective effect of dapsone on cognitive impairment induced by propofol involves hippocampal autophagy. Neurosci. Lett. 649, 85–92. 10.1016/j.neulet.2017.04.019 28411068

[B77] Yirmiya R. Goshen I. (2011). Immune modulation of learning, memory, neural plasticity and neurogenesis. Brain Behav. Immun. 25, 181–213. 10.1016/j.bbi.2010.10.015 20970492

[B78] Youm Y. H. Grant R. W. McCabe L. R. Albarado D. C. Nguyen K. Y. Ravussin A.. (2013). Canonical Nlrp3 inflammasome links systemic low-grade inflammation to functional decline in aging. Cell Metab. 18, 519–532. 10.1016/j.cmet.2013.09.010 24093676 PMC4017327

[B79] Youm Y. H. Nguyen K. Y. Grant R. W. Goldberg E. L. Bodogai M. Kim D.. (2015). The ketone metabolite beta-hydroxybutyrate blocks NLRP3 inflammasome-mediated inflammatory disease. Nat. Med. 21, 263–269. 10.1038/nm.3804 25686106 PMC4352123

[B80] Zhang M. Deng Y. N. Zhang J. Y. Liu J. Li Y. B. Su H.. (2018). SIRT3 protects rotenone-induced injury in SH-SY5Y cells by promoting autophagy through the LKB1-AMPK-mTOR pathway. Aging Dis. 9, 273–286. 10.14336/ad.2017.0517 29896416 PMC5963348

[B81] Zhang X. Dong H. Li N. Zhang S. Sun J. Qian Y. (2016). Activated brain mast cells contribute to postoperative cognitive dysfunction by evoking microglia activation and neuronal apoptosis. J. Neuroinflammation 13:127. 10.1186/s12974-016-0592-9 27245661 PMC4888609

[B82] Zhang Y. Xu Z. Wang H. Dong Y. Shi H. N. Culley D. J.. (2012). Anesthetics isoflurane and desflurane differently affect mitochondrial function, learning and memory. Ann. Neurol. 71, 687–698. 10.1002/ana.23683 22368036 PMC3942786

[B83] Zheng B. Lai R. Li J. Zuo Z. (2017). Critical role of P2X7 receptors in the neuroinflammation and cognitive dysfunction after surgery. Brain Behav. Immun. 61, 365–374. 10.1016/j.bbi.2017.01.005 28089560 PMC5316360

[B84] Zhou R. Yazdi A. S. Menu P. Tschopp J. (2011). A role for mitochondria in NLRP3 inflammasome activation. Nature 469, 221–225. 10.1038/nature09663 21124315

[B85] Zou J. Chen Z. Liang C. Fu Y. Wei X. Lu J.. (2018). Trefoil factor 3, cholinesterase and homocysteine: potential predictors for Parkinson’s disease dementia and vascular parkinsonism dementia in advanced stage. Aging Dis. 9, 51–65. 10.14336/AD.2017.0416 29392081 PMC5772858

[B86] Zou J. Chen Z. Wei X. Chen Z. Fu Y. Yang X.. (2017). Cystatin C as a potential therapeutic mediator against Parkinson’s disease via VEGF-induced angiogenesis and enhanced neuronal autophagy in neurovascular units. Cell Death Dis. 8:e2854. 10.1038/cddis.2017.240 28569795 PMC5520899

